# What Do Young Infants Do During Eye-Tracking Experiments? IP-BET – A Coding Scheme for Quantifying Spontaneous Infant and Parent Behaviour

**DOI:** 10.3389/fpsyg.2020.00764

**Published:** 2020-04-28

**Authors:** Przemysław Tomalski, Anna Malinowska-Korczak

**Affiliations:** Neurocognitive Development Lab, Institute of Psychology, Polish Academy of Sciences, Warsaw, Poland

**Keywords:** eye-tracking, infant, body movement, embodiment, visual attention, dyadic interactions, self-regulation, sex differences

## Abstract

Eye-tracking measurement of looking is the fundamental method in infancy research. Over the last few decades it has provided many of the most significant discoveries in developmental psychology. Infants engage in looking tasks and use their bodies for learning differently from adults, yet, the breadth of their behavioural repertoire and the constraints that the testing situation places on them remain under-explored. Young infants are tested in close physical proximity to their parent, interact during the experiment and rely on the parent to stay engaged in the task. Infants may also engage a different set of skills (e.g. when self-regulating) to perform the very same looking tasks in comparison with adult participants. We devised a coding scheme to systematically analyse task-relevant (attention to the screen) and extraneous behaviours [body movement, self-touch, non-nutritive sucking (NNS), affect] that infants exhibit during an eye-tracking session. We also measured parental behaviours (attention to the screen or the child), including dyadic interactions with the infant (talking, physical contact). We outline the rationale for the scheme and present descriptive data on the behaviour of a large group of typical 5- and 6-month-olds (*n* = 94) during three standard eye-tracking tasks in two seating arrangements. The majority of infants showed very high and consistent within-group attention to the screen, while there were large individual differences in the amount of limb and body movement and the use of self-regulatory behaviours (NNS, self-touch, object manipulation). Very few sex differences were found. Parents spent most time attending to the screen, but engaged in some forms of dyadic interaction, despite being given standard task instructions that minimise parental interference. Our results demonstrate the variability in infants’ extraneous behaviours during standard eye-tracking despite comparable duration of attention to the screen. They show that spontaneous interactions with the parent should be more systematically considered as an integral part of the measurement of infant looking. We discuss the utility of our scheme to better understand the dynamics of looking and task performance in infant looking paradigms: those involving eye-tracking and those measuring looking duration.

## Introduction

The use of eye-trackers has revolutionised developmental psychology, leading to significant theoretical and methodological developments. Modern, self-calibrating eye-trackers allowed psychologists to continuously monitor infant eye movements even during the first months of life, while leaving the participant free to move and observe either a live presentation or the stimuli on a computer screen. Both macro- and micro-scale visual behaviours can be monitored even under different seating arrangements (car seats or high chairs, or seated on a parent’s lap), also in a variety of settings (in the lab, early intervention centres, homes). The flexibility of eye-tracking methods has lead to a considerable increase in the variability of testing contexts (e.g. testing outside of laboratories), also increasing the potential variability of the infant and the caregiver behaviour during the testing session.

Despite many similarities between the infant and the adult participant in eye-tracking experiments, there are important differences that render eye-tracking experiments with infants a different situation altogether. Infant behaviour cannot be controlled by verbal instructions, experimental paradigms are designed to afford behaviours desired by the experimenter, while the co-operating caregiver is constantly monitoring the situation and assisting with the testing. To our knowledge, the uniqueness of infant eye-tracking testing as a situation and its relation to the infant behavioural repertoire has remained largely unexplored. While several reports discuss various challenges of using eye-tracking in non-standard lab settings (Ballieux et al., 2016), or consider the effects of seating arrangements on task performance (Hessels et al., 2015) or protocol optimisation (Hessels and Hooge, 2019), research to date has not explained the role of diverse situational constraints on infant behaviour, or the variability in seemingly task-irrelevant behaviours. All these factors may contribute to individual differences in performance on visual attention tasks.

### Theoretical Underpinnings

Several independent strands of work suggest that seemingly irrelevant behaviours of young infants during looking tasks are associated with the dynamics of attention allocation and learning. Extensive review of this work is beyond the scope of this paper, but we focus on three selected research themes that informed our coding scheme: (1) embodied attention; (2) emerging self-regulation; (3) infant-parent interactions. We focused on these themes to more comprehensively describe the ecology of infant and parent behaviour during looking tasks. Below we summarise our main goals and link them to existing theoretical and empirical work.

Embodiment of cognition refers to bodily constraints on brain and cognitive processes (e.g. Clark, 1997; Smith and Gasser, 2005). Embodied approach recognises the role of actions in the environment for information processing and their dependence on the biomechanics of the body. This aspect is particularly useful when considering the effects of motor development on infant attention with respect to oculomotor control, postural changes or locomotion. First, voluntary control of eye movements undergoes rapid improvements within the first 6 months of life, so that infants can engage and disengage attention according to their interest and internal goals (e.g. Johnson and Tucker, 1996). However, attention shifting during the first months engages their entire body in order to initiate a saccade, thus looking at the stimuli is coupled with limb movements (Robertson et al., 2001). Moreover, healthy infants engage their entire body in many (if not most) motor actions. This is illustrated by the phenomenon of motor overflow in unilateral reaching tasks, where infants produce extraneous movements of limbs that are not involved in a given action (Soska et al., 2012). In typical development motor overflow declines towards the end of the first year of life (D’Souza et al., 2017). Altogether, these results suggest that infants may produce limb and torso movements during looking tasks, which are likely related to their looking. To date these body movements have rarely been systematically studied.

In the middle of the first year of life infants gradually show a number of behaviours that can be considered self-regulatory: they control incoming sensory stimulation, reduce arousal or regulate affect. While there is extensive literature on the development of self-regulation during infant-parent interactions, less is known about the presence of self-regulatory behaviours during looking tasks. To systematically measure such behaviours, we focused on two proposed mechanisms for reducing arousal and negative affect that are present from around 4 months of age: self-distraction and self-comforting (Ekas et al., 2013). Self-distraction involves active disengagement from the stimulus (i.e. looking away from the screen). Self-comforting involves self-touch or thumb-sucking. Although rarely studied in learning tasks, both kinds of mechanisms are likely helping infants to perform visual perception tasks and affect their looking.

Finally, the testing situation involves not only the infant, but also the parent, who is constantly present and active, even if task instructions seek to minimise parental interference. Multiple theoretical accounts can be used to consider the infant behaviour during visual perception tasks in the context of infant-parent interactions, focusing on regulation (Feldman, 2009; Parsons et al., 2010) or socio-communicative behaviours (e.g. Striano and Reid, 2006; Reddy, 2008). Moreover, specific interactive behaviours that occur while the infant attends to a stimulus may modulate learning or create an ongoing communicative context that is associated with learning. Parental affective touch during face-to-face interactions facilitates looking at the parent (Provenzi et al., 2020), but in a visual perception task it enhances discrimination of novel faces in 4-month-olds (Della Longa et al., 2019). Meanwhile, looking at the parent may serve as a better measure of learning in a violation of expectation task than looking at the screen itself (Dunn and Bremner, 2017). These results suggest a need for more systematic analysis of interactive behaviours during visual perception tasks in infancy, especially with respect to infant looking at the parent, the distribution of parental looking during the task and the episodes of dyadic touch.

### Motivation for the Coding Scheme, Goals and Research Questions

In this article we outline a novel scheme for coding and quantifying a range of behaviours during a typical testing session in order to explore the dynamics of looking, movement and self-regulation as infants perform eye-tracking tasks. We focus on presenting this coding scheme as a useful method for better understanding the behavioural dynamics of infant learning, thus we present data on coded categories, reliability of coding and descriptive data that was obtained. Infants performed three standard eye-tracking tasks, involving attention shifting, visual habituation and free viewing of dynamic and static stimuli. Apart from coding the infant behaviour, we also focused on key parental behaviours, such as their attention to the screen or interactive behaviours, like pointing and talking to the infant.

Our primary goal was to quantify the typically occurring infant and parent behaviours during eye-tracking, measuring the total duration, frequency and mean duration of an instance of that behaviour. To this end we coded a diverse set of categories and tested the reliability of such a coding in a large group (*n* = 94) of typically developing 5- to 6-month-old infants. We coded behaviours that are considered critical for eye-tracking measurement (e.g. looking at the screen or away from it), as well as those considered extraneous/irrelevant to it [e.g. infant body movement, object manipulation, non-nutritive sucking (NNS) or self-touch]. Apart from measuring these individual behaviours, we intended our coding scheme to capture the episodes of infant-parent dyadic interactions and various parental behaviours focused on the infant looking that are possibly significant for task performance, but rarely considered task-relevant. Thus, our study allowed for a more systematic exploration of infant looking at the parent, dyadic touch, parental talking and pointing to the screen – a set of interactive behaviours that may occur during an infant eye-tracking session, but rarely have been investigated in this context.

Our second goal was to explore the between-subject variability in these behaviours. Measuring both duration and frequency of behaviours allows to establish the consistency of some behaviours (e.g. variability in duration of looking at the screen), as well as the presence or absence of task-irrelevant behaviours that may help the infant to focus on the task or to modulate their arousal (e.g. self-touch, NNS, object manipulation). Also, given that differences in the level of motor activity are associated with attention shifting in the first months of life (Robertson et al., 2001), we hypothesised that 5- and 6-month olds will show an association between body movement and the duration of attention to the screen.

Our third goal was to systematically test sex differences in infant behaviour as a source of individual differences. Some reports suggest an early presence of sex differences in the level of motor activity (Campbell and Eaton, 1999) or fixation patterns (Lewis et al., 1966). They show ambiguous results for look durations (Creighton, 1984), however, the latter results were obtained for small samples. Since the extent and presence of sex differences in infancy is still debated, we capitalised on our larger sample size to systematically test sex differences in all coded categories. On the basis of previous research we expected greater level of body movement in boys than girls, but did not expect differences in attention to the screen.

Finally, our last goal was to compare the behaviour of the infant and the parent with respect to different seating arrangements: sitting on a parent’s lap vs. in a high chair. Each situation poses different constraints on the infant’s movement. Sitting on a lap engages the parent, who may constantly monitor and adjust the infant’s posture. This constrains infant movements, but provides richer tactile stimulation. Thus, it constitutes a different testing environment than sitting alone in a high chair, even if the parent is seated immediately behind. As seating may systematically affect the infant’s ability to control posture and shift attention, we expected to find more body movement, more looking away from the screen and more self-regulatory behaviours in infants seated in a chair than on a lap.

## Methods

### Participants

The data were collected from a group of 120 infants taking part in a longitudinal study of attention and cognitive development. Here we present data from the first visit at the age of 5–6 months (*M* age = 165.67 days, range 134–200). All participants had normal birthweight (>2500 g), were healthy and were delivered at term (36–42 weeks gestational age). Participants were excluded from analyses due to: fussiness (*n* = 3), calibration problems or eye-tracking equipment error (*n* = 15), or missing/low quality video recording of infant behaviour (*n* = 8). The final sample consisted of 94 healthy infants (47 girls) at the age of 134–189 days (*M* = 166.21; SD = 13.28). Mean maternal age at infant birth was 30.60 years (SD = 3.93, range 23–39). The sample consisted of predominantly middle-class families from a city with over 1.5 million inhabitants. Maternal education was on average *M* = 17.26 of completed years (SD = 1.84, range 11–24). The study was approved by the local institutional ethics committee. All parents gave written informed consent before testing and received a small gift (baby book) and a certificate of attendance.

### Procedure

After a brief warm-up time in the babylab testing room, infants were seated in a high chair (*n* = 20) or on a parent’s lap (*n* = 74) approximately 60 cm from the monitor. The seating was decided by the parent at the beginning of the session. If an infant was seated in a high chair (with additional padding to make it suitable for this age group), the parent sat in a chair positioned to the left and just behind the high chair in order to touch and soothe the infant, if necessary.

Eye-tracking data were collected on a Tobii T60XL eye-tracker (Tobii Inc.) with a 24″ monitor, 60 Hz sampling rate and 0.5° accuracy (value provided by the manufacturer). The five-point infant-friendly calibration was applied. Experimental tasks were presented after the infant successfully calibrated at least four points. Infants performed four blocks of each of the three gaze-contingent tasks (gap-and-overlap, habituation and free viewing; for task description see Wass and Smith, 2014; Tomalski et al., 2017; Niedźwiecka et al., 2018). The blocks of the tasks were interweaved and presented in two pseudorandom orders. The duration of the entire eye-tracking session (including calibration) did not exceed 10 min. For brevity, we do not describe these tasks here in detail, as the eye-tracking data analysis was not the goal of the present report and we analysed the entire session, including very short breaks between experimental blocks.

Infant behaviour was recorded by a remote-controlled CCTV camera (SD image quality) and a microphone placed on a wall approximately 1 m above the stimulus monitor (slightly to the left). Except for the monitor and camera lens, the entire area around the eye-tracker monitor was covered with a black cloth to provide a uniform background. The camera view was set to record the entire body of an infant together with a view of at least the upper part of the parent’s body. The recording started when the first task began and lasted until the session was terminated.

### Coding Scheme for Measuring Infant and Parent Behaviour During Eye-Tracking

#### Development of the Coding Scheme

To measure the duration and frequency of behaviours exhibited by infants and parents during eye-tracking sessions we developed a new coding scheme. We focused on constructing categories that may capture the variability and dynamic nature of infant behavioural repertoire that has rarely been systematically studied in eye-tracking research. The categories of behaviours were selected on the basis of in-depth observation of recordings of pilot eye-tracking sessions. The review and selection of categories for coding was also supplemented by the existing literature on the microanalysis of infant behaviour during interactions (e.g. Feldman et al., 1999; Beebe, 2006), while in the case of movement we incorporated categories derived from global rating scales of interactions (Wan et al., 2013). The coding scheme was developed in two stages. The usability and reliability of the initial set of 37 behaviours arranged in eight categories was tested in a study of 30 infants aged 5 and 9 months and resulted in the rejection of very rare and low-reliability categories. The revised set of categories, with corrected definitions was tested on another group of 24 five-month-olds, showing sufficient reliability (Kostecki et al., 2014). Here we present the final version of the scheme, which was used to code a large sample of 5- to 6-month-old infants. For the infant, we coded the following categories: visual attention (looking at screen; aside; at parent), body movement (low, partial, or full movement, excluding head movements), affect (neutral, negative, positive) and other behaviours [NNS, self-touch, object-related activity (ORA)]. For the parent, we coded visual attention (looking at screen, aside, at infant) and other, interactive behaviours (giving something to infant, talking to infant, pointing to the screen). Moreover, we created a dyadic category of physical contact (a state event), which captured joint activity of the infant and the parent to establish physical contact. Full definitions are presented in the Supplementary Table M1.

#### Coding Procedures

Videos were coded in Observer XT11.5 (Noldus Inc.) by four trained coders (undergraduate psychology students), who did not participate in the initial development of the coding scheme. Training involved a short session explaining the definitions of each category and coding three pre-selected recordings. Each coder was deemed trained if satisfactory reliability was obtained for these recordings (Cohen’s kappa >0.8 for movement and looking between trainee and the trainer). We were able to code infant, parent and dyadic categories for all infants seated on a parent’s lap (*n* = 74), but for those seated in a high chair (*n* = 20) in some videos the parent stayed out of the camera’s view for most of the session, thus parental and dyadic behaviours were not coded for a subgroup of participants (*n* = 8). Moreover, seating arrangements may have systematically influenced infant behaviour, so for clarity we first present analyses of the data for infants seated on the parent’s lap (*n* = 74), while the figures present distribution for both seating arrangements. Full descriptive data in table format for infants seated in a high chair are presented in the Supplementary Material, while statistical comparisons between lap and chair seating are summarised at the end of the main results section. In the case of parental and dyadic codes for chair-seated infants because of the lower number of available data points we provide the descriptive data in the Supplementary Material, but did not analyse them statistically, except for comparisons between seating arrangements.

We coded the entire eye-tracking session from the moment the calibration was completed to the end of the session (duration *M* = 582.15 s, SD = 115.41, min = 288.03, max = 877.50). The coding was done in several passes, upon each pass separately coding infant attention, movement, other behaviours, affect, parental behaviour and dyadic physical contact. Video speed for each coded category is provided with the definitions (see Supplementary Table M1). The estimated coding time per participant was 2–3 h.

#### Reliability

In order to establish the inter-rater reliability, 10.8% (*n* = 8) of the videos were second-coded by a trained researcher. Each category was examined separately in terms of correct sequence and duration of each instance. Reliability rates (Cohen’s kappa) were computed using Observer XT (see Supplementary Table S1 for statistics for each category). The scores indicate high level of inter-rater reliability for all categories: (a) infant movement: *M* = 0.870; (b) infant visual attention: *M* = 0.96; (c) infant behaviours-other (NNS, self-touch and object manipulation): *M* = 0.994; (d) infant affect: *M* = 0.991; (e) parent visual attention: *M* = 0.896; (f) dyadic physical contact: *M* = 0.896.

### Data Analyses

For start-stop events we used three main metrics – the total duration throughout the session that the infant showed this behaviour (expressed as proportion of total observation time), the mean duration of an episode in seconds and the frequency of episodes (calculated as rate per minute, that is the mean number of episodes per minute of the observation). For state events we report frequency only (rate per minute).

There were no correlations of coded behaviours with infant age, except for weak, but statistically significant correlations of the total duration of low movement (*r* = 0.27, *p* = 0.018) and total duration of infant looking at the parent (*r* = 0.24, *p* = 0.041). For these reasons we omitted participant age from subsequent analyses, but note that this could be the result of a limited age range in the sample (spanning approximately 2 months).

Our main analyses of movement and visual attention used mixed-model repeated-measures ANOVAs with behaviour category as a within-subject factor and participant sex as a between-subject factor. Since nearly all analyses did not return a significant main effect of sex or an interaction, for brevity we do not report these null results. Greenhouse – Geisser correction was used, where appropriate. All pairwise comparisons for ANOVA results were Bonferroni-corrected. Where necessary, additional group comparisons were conducted using non-parametric Mann–Whitney test.

Associations between infant movement and looking were tested with Pearson correlations, while comparisons of group means between infants seated in a chair or on a lap used independent *t*-tests, which are robust to large discrepancies in group size.

## Results

### Infant Behaviours

#### Body Movement

**FIGURE 1:**
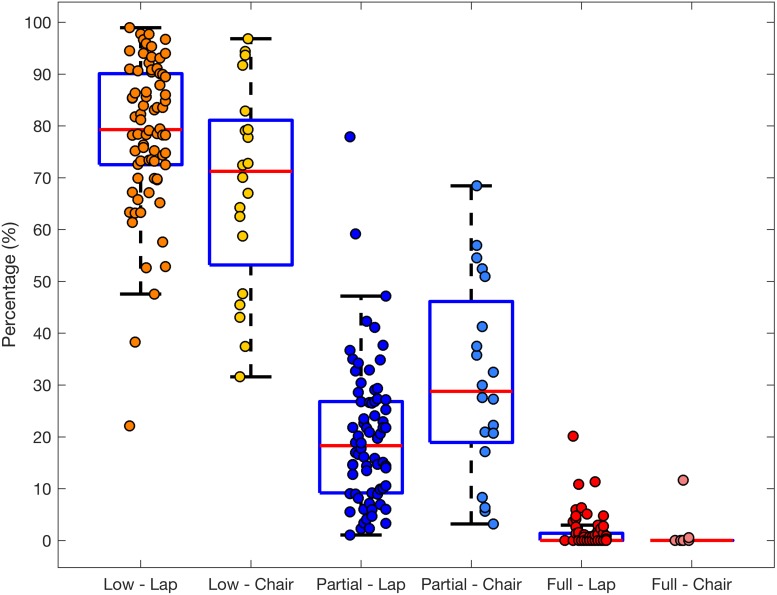
Boxplot with overlaid dots (individual participants) representing the total duration (proportion of observation time) of low, partial and full movement for infants seated on a lap (*n* = 74) or a chair (*n* = 20).

During the entire session infants spent the majority of time in low body movement (*M* = 78.59, SD = 14.40). The remaining observation time was spent mostly in partial body movement (*M* = 19.97, SD = 13.61) with the duration of full movement (all limbs and torso moving) lasting on average for less than 2% of time (*M* = 1.44, SD = 3.15). These differences in total duration were significant for the entire group [3 × 2 ANOVA, main effect of movement category, *F*(2,144) = 590.06, *p* < 0.001, $\eta_{p}^{2}=0.89$]. See Figure 1 for the distribution and Supplementary Table S2 for total and mean duration and frequency by participant sex.

Likewise, the episodes of low movement were on average longer (*M* = 25.49, SD = 21.84) than those of partial (*M* = 4.82, SD = 3.30) or full movement [*M* = 1.74, SD = 2.34, main effect of movement category, *F*(2,144) = 70.16, *p* < 0.001, $\eta_{p}^{2}=0.49$]. Over 50% of infants of both sexes exhibited episodes of full movement, and they were on average shorter than episodes of partial movement (*p* < 0.001), which, in turn, were shorter than episodes of low movement (*p* < 0.001).

The frequency data resembled that of duration, with a significant main effect of movement category [*F*(2,144) = 315.16, *p* < 0.001, $\eta_{p}^{2}=0.81$]. Low movement episodes were more frequent (*M* = 2.59, SD = 1.13) than episodes of partial movement (*M* = 2.51, SD = 1.14, *p* < 0.045), which in turn were more frequent than full movement episodes (*M* = 0.21, SD = 0.4, *p* < 0.001).

#### Visual Attention

**FIGURE 2:**
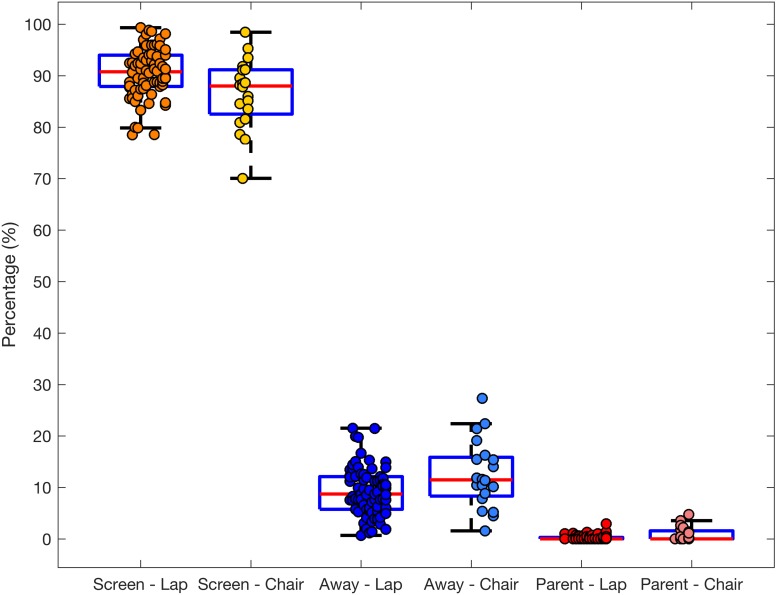
Boxplot with overlaid dots (individual participants) representing the total duration of looking at the screen, away from it or at the parent for infants seated on a lap (*n* = 74) or a chair (*n* = 20).

First, we analysed the duration and frequency of infant looking at the screen and away from it with a 2 × 2 mixed-model ANOVA (looking at the screen vs. away × participant sex). Next, we separately analysed the infrequent episodes of looking towards the parent. See Figure 2 for the overall distribution and Supplementary Table S3 for averages broken down by participant sex.

All infants spent the vast majority of time looking at the screen (*M* = 90.50, SD = 4.72) rather than looking away from it [*M* = 9.26, SD = 4.69; main effect of looking category, *F*(1,72) = 5649.80, *p* < 0.001, $\eta_{p}^{2}=0.98$]. There were no significant sex differences in the total duration of looking [participant sex × looking category, *F*(1,72) = 3.23, *p* = 0.077, $\eta_{p}^{2}=0.43$], with boys showing a tendency to look shorter (*M* = 89.49, SD = 4.94) at the screen than girls (*M* = 91.40, SD = 4.39). However, these differences were very small in magnitude and amounted to approximately 2% of the observation time.

The mean duration of an episode of looking at the screen was also significantly longer (*M* = 19.67, SD = 18.36) than duration of an episode of looking away from it [*M* = 1.57, SD = 0.61; main effect of looking category, *F*(1,72) = 70.18, *p* < 0.001, $\eta_{p}^{2}=0.49$].

Approximately 40% of infants looked towards the parent during the session, but these episodes were very short, on average just over 1 s in duration (*M* = 1.19, SD = 0.67) and occurred only a few times throughout the session (*M* = 0.30, SD = 0.25).

#### Other Behaviours

##### Non-nutritive sucking

**FIGURE 3:**
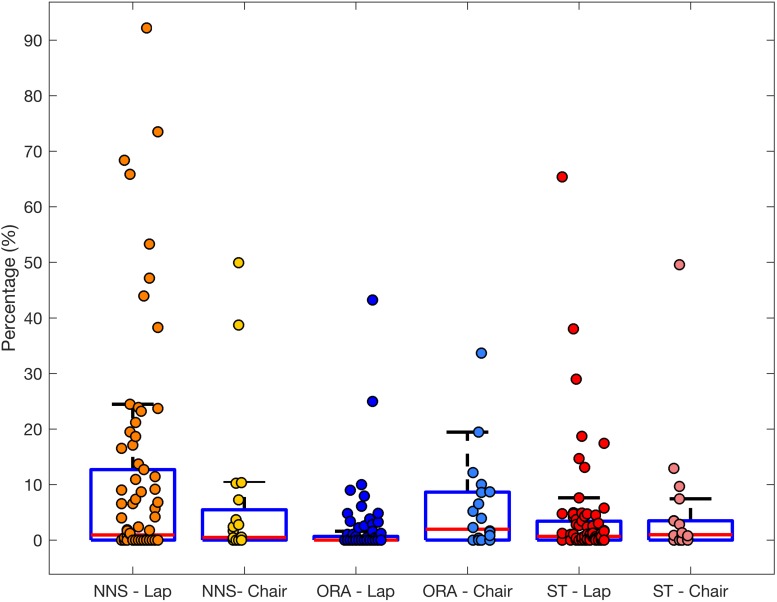
Boxplot with overlaid dots (individual participants) representing the total duration of non-nutritive sucking (NNS), object-related activity (ORA) and self-touch for infants seated on a lap (*n* = 74) or a chair (*n* = 20).

While performing eye-tracking tasks infants engaged in a range of oral and manual behaviours. Oral behaviours included NNS on own fingers or moving them inside the mouth. Parents were free to decide whether to provide the infant with a pacifier, but only five infants were given one. Since the majority of NNS involved own fingers rather than external objects, we calculated duration and frequency collapsed for pacifier and non-pacifier users. On average these behaviours were present for nearly 20% of time (M = 19.97, SD = 22.95), with each episode lasting M = 38.33 s (SD = 73.72) and occurring less than once per 2 min (*M* = 0.47, SD = 0.39). Two-thirds of boys and less than half of girls showed NNS (χ^2^ = 3.67, *p* = 0.055). There were no sex differences in either total or mean duration of sucking episodes or in their frequency (all *p*s > 0.156). See Figure 3 for the frequencies and Supplementary Table S4 for group averages broken down by participant sex.

##### Object-related activity

Nearly a quarter of boys and a third of girls showed ORA (no significant sex differences, χ^2^ = 0.86, *p* = 0.35). The vast majority of behaviours involved infants manipulating and holding their clothes or parent’s clothes with their hands. We did not observe any sex differences in the duration of these episodes (both *p*s > 0.54), but boys (*M* = 0.45, SD = 0.27) manipulated objects more often than girls (*M* = 0.27, SD = 0.22; *U* = 24.00, *p* = 0.045).

##### Self-touch

Nearly three quarters of boys and just over half of girls showed self-touching behaviours during eye-tracking (approaching significance trend, χ^2^ = 3.06, *p* = 0.08; see Supplementary Table S4). Among the infants that showed these behaviours boys (*M* = 9.29, SD = 14.96) spent disproportionately more time exhibiting self-touch than girls (*M* = 2.51, SD = 3.75; *U* = 162.0, *p* = 0.027). There was a non-significant trend for boys to show more frequent (*U* = 181.5, *p* = 0.074) and longer episodes of self-touch (*U* = 182.0, *p* = 0.076) than girls.

##### Regulatory behaviours and attention to the screen

We compared infants who showed specific regulatory behaviours and those that did not to test whether it was associated with differences in attention to the screen. Infants showing NNS (*n* = 40) looked equally long at the screen as those that did not [*t*(72) = 1.75, *p* = 0.085]. No differences were also found for infants showing object manipulation (*n* = 21) in comparison to those that did not [*t*(72) = 0.15, *p* = 0.88]. By contrast, participants that used self-touch (*n* = 46) during the session (*M* = 89.37, SD = 4.64) looked significantly less at the screen than those that did not [*M* = 92.35, SD = 4.34; *t*(72) = 2.75, *p* = 0.008], although this difference amounted to only 3% of the entire session time.

#### Affect

Infants showed neutral affect for the majority of testing session time, and there were no sex differences in the overall neutral affect duration [*t*(72) = 1.6, *p* = 0.114; see Supplementary Table S4]. However, boys showed higher variability in changes of affect than girls, which was illustrated by differences in mean duration of a neutral affect episode [*t*(72) = 2.42, *p* = 0.018]. The were no sex differences in the proportion of participants showing positive or negative affect (both *p*s > 0.29). Likewise, there were no sex differences in the duration of episodes of either positive or negative affect (both *p*s > 0.35).

Previous research indicated that the primary function of self-comforting behaviours is to reduce negative affect, so we compared their frequency and duration for infants, who showed any negative affect during the session (*n* = 22) and those that did not (*n* = 52). There were no differences in either total and mean duration or frequency of NNS (all *U*s < 688, all *p*s > 0.15), as well as object manipulation (all *U*s < 678, all *p*s > 0.12). By contrast, infants that expressed negative affect showed higher frequency of self-touch episodes relative to infants that did not show negative affect (*U* = 770.5, *p* = 0.016). They also showed longer total duration (*U* = 770.0, *p* = 0.017) and a near-significant trend for mean duration of self-touch (*U* = 727.5, *p* = 0.06).

### Parental Behaviours

#### Parental Visual Attention

**FIGURE 4:**
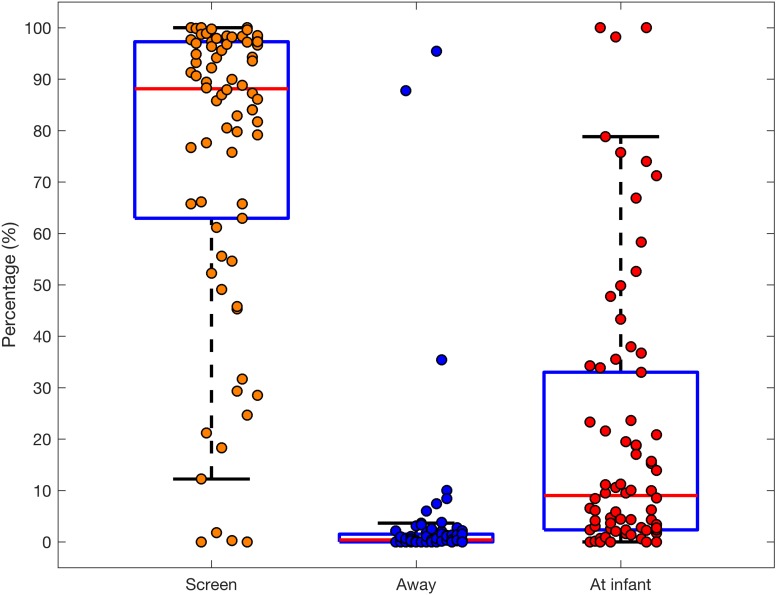
Boxplot with overlaid dots (individual participants) representing the total duration of the parent looking at the screen, away from it or at the infant (only parents with infant on a lap, *n* = 74).

For the majority of observation time parents attended to the screen, while the infants were performing eye-tracking tasks – on average they looked at the screen for more than 75% of the entire time (*M* = 75.44 SD = 29.40), with each episode lasting more than a minute (*M* = 69.54 SD = 83.55; see Figure 4 and Supplementary Table S5 for full data). The remaining time was spent mostly looking at the infant (*M* = 20.40 SD = 26.52), but these events were considerably shorter, lasting on average nearly 30 s (*M* = 29.58 SD = 104.71) Parents spent relatively little time looking away from the screen, only around (*M* = 4.04 SD = 15.36) of the time, and these were relatively short glances, taking on average just over 7 s (*M* = 7.19 SD = 29.46). These differences were significant at group level for both total [χ^2^(2) = 99.92, *p* < 0.001] and mean duration [χ^2^(2) = 81.53, *p* < 0.001] and frequency [χ^2^(2) = 101.79, *p* < 0.001].

#### Other Parental Behaviours – Pointing and Talking

We also coded other parental behaviours that occurred during the testing session (see Supplementary Table S5). Nearly half of parents (*n* = 34, 45.9%) holding infant on their lap talked to him/her. A qualitative review of these events revealed that these most commonly were comments on the content of the task, infant seating and focus of attention or infant state during the session. Likewise, nearly a quarter of parents (*n* = 19, 25.7%) were occasionally pointing to the screen, typically to bring the infant’s attention back to the stimuli.

### Dyadic Physical Contact

Episodes of dyadic physical contact were coded, when both the infant and the parent were actively engaged in movements that established or maintained specific forms of physical contact. They included grasping and holding the infant’s hand, cuddling or hugging. They also included activities initiated by the infant, e.g. holding the parent’s hand or finger or manipulating the parent’s clothes to seek physical contact (see Supplementary Table S6 for full data). The vast majority of infant-parent pairs (*n* = 64, 86.5%) exhibited such dyadic activity. On average the dyads remained in active physical contact for nearly 20% of testing session time (*M* = 18.37, SD = 22.63), while each episode lasted less than half a second, although we recorded high variability in average duration (*M* = 23.93, SD = 74.08). The average frequency was below 1 event per second (*M* = 0.86, SD = 0.67).

### Associations Between Infant Looking Away From the Screen and Body Movement

**FIGURE 5:**
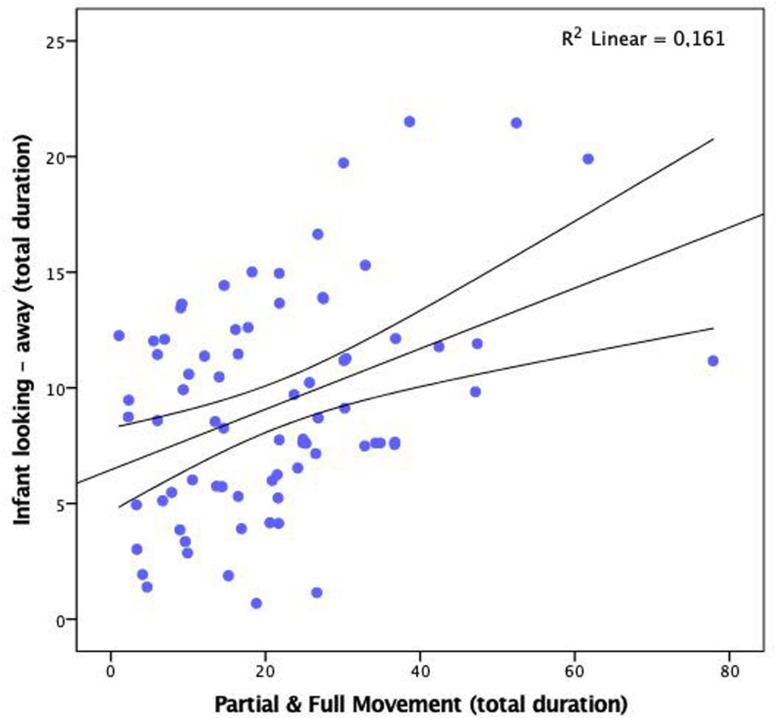
Scatterplot showing correlation between the duration of partial and full movement and the duration of looking away from the screen (infants seated on a lap, *n* = 74). Duration given as percentage of total observation time.

Infant body movement was significantly associated with looking at the screen or away from it (see Figure 5 and Supplementary Table S7). The total duration of time that infants exhibited partial or full body movement was negatively correlated with the total duration of looking at the screen (*r* = −0.396, *p* < 0.001) and positively with looking away from it (*r* = 0.400, *p* < 0.001). Also, infants that engaged in more body movement, moving their torso and limbs, were more likely to shift their attention between the screen and the surroundings. We found positive correlations of the total duration of partial and full movement with the frequency of attention shifting towards (*r* = 0.362, *p* = 0.002) and away from the screen (*r* = 0.362, *p* = 0.002). Infant movement was not significantly associated with the duration of episodes of looking at the screen or away from it.

For those infants that looked at the parent (*n* = 30) we also tested the associations between body movement and looking at the parent. We did not find any correlations between total duration of partial and full movement the total and mean duration or frequency of episodes of looking at the parent (all *r*s < 0.25, all *p*s < 0.18). Thus, while we found that individual differences in body movement were related to infant looking towards and away from the screen, we did not find any evidence for associations of movement with infant attention to the parent during the session.

### Effects of Seating Arrangements on Infant Behaviour

#### Body Movement

Infants seated in a high chair moved significantly more than those seated on a lap. They showed higher total duration of partial movement [*M* = 30.97, SD = 18.78 vs. *M* = 19.97, SD = 13.61, *t*(24.65) = −2.45, *p* = 0.022], longer average duration of partial movement episodes [*M* = 8.51 SD = 5.45 vs. *M* = 4.82, SD = 3.30, *t*(22.90) = −2.89, *p* = 0.008] and correspondingly lower duration of low movement [*M* = 23.06, SD = 16.43 vs. *M* = 25.49, SD = 21.84, *t*(24.82) = 2.17, *p* = 0.04]. We did not observe any differences in the frequency of episodes of movement (all *p*s > 0.14).

#### Visual Attention

Total duration of looking differed depending on seating arrangements. Infants seated in a highchair on average looked somewhat less on the screen [*M* = 86.58, SD = 6.67; *t*(92) = 2.99, *p* = 0.004] and looked away from the screen for longer [*M* = 12.51, SD = 6.55; *t*(92) = −2.51, *p* = 0.014] than infants on a lap (screen: *M* = 90.50, SD = 4.72 and away: *M* = 9.26, SD = 4.69). There were no significant differences in the mean episode duration (both *p*s > 0.18) or frequency of looking towards or away from the screen (both *p*s > 0.08).

The proportion of participants that were looking towards the parent was comparable across both seating arrangements (lap: *n* = 30, 40.5%; chair: *n* = 8, 40%). However, those seated in a high chair looked for longer periods of time [*M* = 2.76 SD = 3.17, *t*(7.62) = −3.11, *p* = 0.015] and more often [*M* = 0.62, SD = 0.32, *t*(36) = −3.04, *p* = 0.004] than those seated on a lap [*M* = 1.19, SD = 0.67 and *M* = 0.30, SD = 0.25, respectively].

#### Other Behaviours

We did not find any differences between the chair and lap infants in the duration or frequency of NNS, ORA, or self-touch (all *p*s > 0.19).

## Discussion

Infant behaviour during looking tasks, especially those using eye-tracking, is very different from the behaviour of typical adults in such experiments. This is partly related to differences in embodied cognition between infants and adults. Moreover, infant eye-tracking research often deals with low-quality and noisy data (Wass et al., 2014), due to limited controllability of participant behaviour. Exploring the sources of variability in infant behaviour during eye-tracking is important for understanding how they perform individual tasks, e.g. in terms of associations between body movement and task performance. In our study we devised a coding scheme to explore the dynamics of looking, movement and self-regulatory behaviours as infants performed three standard, gaze-contingent eye-tracking tasks. Our goal was to more comprehensively describe and quantify the range of behaviours that 5- to 6-month-olds exhibit during eye-tracking, including those often considered irrelevant to task performance. We also investigated several parental behaviours that have rarely been studied and quantified during infant eye-tracking, such as parental attention to the stimuli or pointing to the screen, together with selected dyadic episodes of active touch between the infant and the parent. The scheme categories proved easy to use and allowed very high inter-rater reliability for all categories.

### Attention to the Screen and Body Movement

During a standard eye-tracking session our participants spent most of the time, nearly 90%, attending to the screen, looking away from it for approximately 10% of time. Individual differences in those proportions were relatively low, as one standard deviation amounted to less than 5% of the session duration. Episodes of looking away were relatively short and infrequent. They lasted on average only 1.5 s and occurred approximately 3 times per minute. Less than half of participants occasionally looked towards the parent during the session, thus showing clear tendency to interact with the parent during the session. We discuss this and other aspects of dyadic interactions further on. Altogether, our data show a remarkable consistency of looking behaviour during eye-tracking at a very young age. These results were obtained for a relatively large sample of typically developing infants, across three different tasks that either required rapid orienting to peripheral targets, or free viewing of static and dynamic stimuli. The high consistency with which infants maintained their looking at the screen is even more interesting in the light of the data on movement and self-regulatory behaviours that infants exhibited, suggesting that infants may use a range of additional behaviours to maintain their looking at the stimuli throughout the session.

Even young infants actively engage their entire bodies during cognitive activity and these movements may serve multiple functions (e.g. Doolittle and Ruff, 1998; Robertson et al., 2001). We coded the overall amount of body movement in terms of how many body parts were involved, arriving with three categories: low, partial (only arms or legs and torso) and full movement (all limbs and torso). These categories proved effective in qualitative analyses of infant social interactions (Wan et al., 2013), but we adapted them to quantify the duration of movement. As expected, participants showed long periods of low movement (nearly 80% of the session time), when limbs and torso are inactive. These periods were separated by shorter bouts of more intensive movement of selected limbs and torso, lasting on average 4–5 s. Individual differences in the duration and frequency of partial movement were much higher than in the case of looking. One standard deviation in the duration of partial movement reached nearly 15% of the session time. Also, more than half of the infants exhibited very short and rare episodes of full movement, involving both legs and arms and the torso moving simultaneously. One obvious explanation of these bouts is agitation due to negative affect, however, we found only a weak correlation between the duration of more intensive movement and affect (explaining <9% variance) and only a few infants showed negative affect for more than 3% of session time. While this question requires more in-depth sequential analysis, we tentatively conclude that bouts of body movement may serve other functions than merely expressing affect or high arousal.

Head movements were not included in the body movement category, as they may have depended on attention to the screen, which was analysed separately. Thus our analysis quantified periods of more intensive body movement regardless of the focus of attention and was unlikely confounded by head turns. Our results highlight the discrepancy between the high variability in the duration of body movement and the consistently high looking at the screen across the sample. It may suggest the early onset of individual differences in the association between gross motor activity and oculomotor systems. While all participants looked at the screen most of the session time, some showed minimal movement, but others performed regular limb and torso movements. Increased body movement was not associated with large amounts of looking away from the screen. The correlation between the movement and looking away was significant but small. Movement duration explained only 16% of variance in duration and frequency of looking away from the screen. Partial correlations showed that this association was independent of the duration of negative affect. These results may seem puzzling at first, but appear consistent with previous results with younger infants, aged 1 and 3 months, where short bursts of body movement facilitated attention shifting (Robertson et al., 2001). Further studies by Robertson and Johnson (2009) revealed individual differences in the extent to which infants suppress these bursts after performing an eye movement, which may have long-term consequences for the development of attention control and its disorders (Friedman et al., 2007). One implication of our results is that any physical constraints on body movement during eye-tracking (through restricted seating or parent holding) may differently affect looking times for different infants. Standard procedures should take into account this fact in future eye-tracking studies.

### Other Regulatory Behaviours

At 5 months of age infants show a range of regulatory behaviours, some involving visual disengagement, and others involving self-comforting (Stifter and Braungart, 1995). NNS, object manipulation and self-touch were previously interpreted in this age group as tools for self-soothing, reducing negative affect and arousal (Bruner, 1973; Stifter and Braungart, 1995; Ekas et al., 2013). Our data show that all these behaviours were relatively infrequent, occurring once every 2 min. Whereas some were performed by few infants (object manipulation), others – by the majority (NNS and self-touch). Only a few participants received a pacifier from the parent, but more than 50% of infants showed NNS (mostly own fingers) for approximately 20% of the session time, with average episode lasting relatively long: 38 s. Over 60% of participants exhibited self-touch, which took on average only 6% of the total time, a single episode lasting 7 s. A minority of infants touched or manipulated available objects and these episodes lasted on average 10 s, with the total duration amounting to nearly 6.5% of the session time. Altogether, these results indicate that young infants exhibit a number of self-comforting behaviours during eye-tracking and some may last very long relative to the duration of individual visual stimuli. Previous literature identified the primary role of self-comforting behaviours as reducing negative affect. It was demonstrated in situations of visible distress, e.g. arm restraint (Stifter and Braungart, 1995). Standard eye-tracking test situations do not lead to high levels of distress and we observed only short episodes of negative affect in a small proportion of participants. Moreover, in case of visible distress testing is interrupted or discontinued. However, we tested whether infants, who expressed negative affect were more likely to show these behaviours. Infants who expressed any negative affect during the entire session were more likely to self-touch, and their self-touch episodes were longer and more frequent. We did not find any significant differences with respect to NNS and object manipulation. We conclude that NNS and object manipulation may serve other functions than only self-comforting to reduce negative affect. Moreover, research with toddlers shows that self-touch may also help to regulate attention and not only affect (Ito-Jäger et al., 2017).

During the eye-tracking session NNS and object manipulation (as opposed to self-touch) may help the infant to maintain the appropriate level of arousal, e.g. by generating additional motor activity, as infants try to maintain their looking at the screen. Thus, the presence of these behaviours in 5- and 6-month-olds may actually reflect their individual strategies of maintaining attention to stimuli and attempts to stay in the task. We did not find significant differences in the duration of looking to the screen between infants that show these behaviours and those that did not. By contrast, infants who showed self-touch were looking away from the screen significantly more often and for longer time than those that did not. While the exact function of NNS and object manipulation requires further analysis, our results demonstrate large individual differences in the use of specific self-regulatory behaviours by infants during visual perception tasks. Our coding scheme offers a means of systematically quantifying them to investigate their exact role in modulating task performance.

### Parental Behaviour During the Session

Our coding scheme was useful in exploring free, unconstrained behaviour of parents that sit together with the infant during the session. This aspect of eye-tracking methodology has rarely been considered systematically, beyond controlling parental interference. Typically, the parent is in close physical proximity, monitors the infant’s posture and his/her behaviour in relation to the task, potentially playing a significant role in maintaining attention to the screen and regulating fluctuations of arousal in response to the task. We found that approximately 45% of parents at least once talked to the infant, producing on average more than one utterance per minute. Also, 25% of parents pointed to the screen, on average every 2 min. Thus, even if parents are given task instructions and they are aware of the need to not disrupt the flow of the task – they consider it important to talk to the infant and re-direct attention back to the screen.

The analysis of parental looking revealed that on average they spent around 75% of time attending to the screen. While there was considerable variation (one standard deviation was nearly 30% of total time), they looked at the screen less than their infants. Moreover, parents spent a considerable amount of time looking at their infant throughout the session (20% of time on average) and very little time looking elsewhere. Thus, the typical looking behaviour of a parent having the infant seated on her lap was long looks towards the screen (on average over 60 s), separated by occasional looks at the infant (mean duration ∼30 s) and rare, short looks away from the screen. Altogether, during a typical eye-tracking session parents spent large amounts of time monitoring their infant’s behaviour, occasionally interfering in order to re-direct attention back to the screen or to communicate.

### Dyadic Interactions During the Session

Our final category involved coding the episodes of shared physical contact between the infant and the parent. We considered episodes of physical contact as the key manifestation of an ongoing dyadic interaction during eye-tracking, as visual attention of both partners was engaged primarily with the stimuli on the screen. We labelled such episodes “dyadic” as both partners needed to coordinate their actions to maintain physical contact, regardless of who initiated it. Episodes of dyadic physical contact were relatively long, lasting on average 24 s and they were present in the majority of dyads (over 85%), and on average represented 20% of session time. They were differentiated in our scheme from situations, where the parent merely holds the infant in upright position in front of the screen, but there is no additional interaction that would involve active touch or cuddling. In this way, this dyadic category could be used irrespective of seating arrangements (both lap and chair). Data for infants seated in a chair show comparable total duration, but longer duration and lower frequency of individual episodes, however, these results require replication in a larger sample, as in several cases we were unable to code parental behaviour for this group.

During typical free play of 5-month-olds and parents, the dyadic coordination of visual attention plays a key role in the interaction and predicts later attention control in the infant (Niedźwiecka et al., 2018). In comparison with free play settings, the situation of eye-tracking considerably alters the way in which infants and parents interact. Their attention is focused primarily on the screen, their vocal activity is likely reduced, so maintaining physical contact becomes an important mode of communication. The infant may seek it for a number of reasons (regulation, communication about the task and stimuli) and the parent may signal her/his presence and attention to the infant. Thus, dyadic touch may signal that the infant is the object of the parent’s attention (see Reddy, 2008). Moreover, recent studies suggest that active touch modulates infant looking and stimulus processing (e.g. Della Longa et al., 2019). For this reason, coding and analysing episodes of touch and physical contact may provide important information that explains infant visual behaviour and task performance.

### Sex Differences in Infant Behaviour

One of the surprising results is that we found very limited evidence of sex differences in behaviour of young infants during eye-tracking at the age of 5 to 6 months. This is in contrast to a handful of reports showing sex differences in motor and cognitive skills at this age (e.g. Lewis et al., 1966; Campbell and Eaton, 1999; Moore and Johnson, 2008; Alexander et al., 2009), although some of these effects have not been replicated (Creighton, 1984; Erdmann et al., 2018). Comparisons for individual categories showed only small, statistically significant effects for visual attention. On average boys looked less towards the screen and more away from it than girls. We found some approaching significance trends in self-regulatory behaviours: object manipulating and self-touch were more frequently used by boys than girls. Contrary to available data on early sex differences in motor activity (Campbell and Eaton, 1999), we did not find any differences between boys and girls in the duration or frequency of movement during the session. Altogether, our results may suggest that the existing data on the early emergence of sex differences could be context-specific so that varying conditions in which infant behaviour is measured may affect the observed effect sizes.

### Limitations

Our approach to studying spontaneous infant behaviour during eye-tracking offers several advantages, such as measurement of duration of individual behaviours and the analysis of social-interactive aspects of infant and parent behaviour. But before we discuss the utility of our scheme in the next section, we note some limitations of our method and approach. First, the definition of movement categories allows only for relatively crude measurement of body movement dynamics and does not differentiate different movement types or engagement of individual limbs. Since the primary interest of our analysis was to track overall movement dynamics while infants perform looking tasks, our scheme will be less useful in analyses, where specific motor responses are tracked (e.g. asynchronous activity of individual limbs in reaching tasks). Moreover, it is likely that some of our analyses of individual differences in looking and movement are confounded by differences in gross motor development, for which we did not control, although this criticism could be applied to the majority of existing infant eye-tracking studies. We also note the limitations on the temporal resolution of manual coding, when analysing very short bursts of motor activity, which may require more advanced methods, such as pressure mats (e.g. Reddy et al., 2013).

Second, our current analysis tracked infant and parent spontaneous behaviour during standard test situation. Parents were given standard instructions (i.e. not to talk or interfere with infant looking and behaviour, to maintain infant in a stable and upright position at constant distance from the screen, etc.), but we had limited control over parental compliance with these instructions. Our data on parental activity (e.g. talking to the infant) suggest large variability in compliance, which ought to be addressed more systematically, or at least monitored for each participant. It is possible therefore that some variability can be attributed to differences in parental inhibitory control.

Third, our comparison of infant behaviour depending on seating arrangements may not be sufficiently robust due to large disparities in group sizes. This was dictated by the fact that parents were free to choose the optimal seating conditions for their infant and relatively few opted for a high chair. Thus, our data reflects the actual ecology of eye-tracking testing for infants aged 5 to 6 months, where parents spontaneously decide on how to optimise the situation. We suggest that our results for the effects of seating ought to be validated in a within-subject design with preferably larger and more balanced group sizes.

Fourth, the proposed coding scheme was designed to study behavioural repertoire during eye-tracking testing. The scheme shows excellent inter-rater reliability of coded categories, but so far it has not been validated in relation to the eye-tracking data itself. Thus, our scheme requires more validation work to study the utility of individual categories with respect to eye-tracking data quality and task performance itself. We suggest research plans for such analyses in the next section, while also noting that the scheme could be useful for visual perception studies where look durations are measured that not use eye-tracking. In many cases the ecology of such tasks is very similar to that of eye-tracking studies.

Finally, manual coding of many behavioural categories, some of them at reduced video speed is labour-intensive and costly. For this reason, we deliberately omitted some behaviours, which are relatively infrequent at the age of 5 to 6 months, such as vocalisations. Future applications of our scheme for analyses of older infants and especially toddlers may require more systematic analysis of quantity and quality of vocalisations.

### Future Directions

The infant and parent behaviour during eye-tracking (IP-BET) coding scheme offers a method for systematic monitoring of the dynamics of infant and parent behaviours as they engage in tasks during an eye-tracking experiment. One potential application is for analysing the role of infant body movement and self-regulatory behaviours for their allocation of attention. We believe that IP-BET may prove useful to investigate the associations between infant motor and social-interactive activity and looking time measures. It also allows to investigate the role of these behaviours for lower-level measures of eye movements – the dynamics of individual fixations and visual scanning. Secondly, there is an increasing need to investigate the role of parental activity and the interaction with the parent as the context, in which the infant learns and explores. To this end our coding scheme may facilitate dynamic analyses that seek to link social-interactive episodes during an eye-tracking session with infant learning and visual attention as the task progresses. We suggest that systematic observation of a variety of infant behaviours may help to better understand, how specific actions on the part of the parent facilitate and enhance stimulus processing or attention to some aspects of the task, e.g. parental pointing to the screen or commenting on the task in infant-directed speech. Also, our data indicate that dyadic social interactions are an intrinsic part of infant eye-tracking studies. Understanding the role of these interactions for fluctuations of infant arousal may help to explain the sources of individual differences in task performance. While we appreciate that for many such analyses there is a need to use more precise instruments and methods (e.g. psychophysiological, or monitoring movement with accelerometry), we believe that our coding scheme may offer a low-tech, easy to use alternative that may facilitate this research. Finally, since our scheme uses primarily continuous categories, and measures behaviour duration, it is useful for dynamical analyses that quantify the inter-relations between multiple behaviours of two interacting partners, or between the infant and individual stimuli on the screen. Thus, it is highly useful for analyses of coordination between two or more time-series of behaviours, either within infant or between the infant and the parent, such as the cross-recurrence quantification analysis (CRQA; Coco and Dale, 2014), which has been applied to investigate complex dynamics of social interactions or the dynamics of scanning, even in infants (e.g. López Pérez et al., 2017, 2018).

## Conclusion

We present a novel coding scheme for measuring frequency and duration of infant and parent looking, motor and regulatory behaviours as the infant performs standard laboratory eye-tracking tasks. Our scheme shows high inter-rater reliability with 5- and 6-month-olds and can be used under different seating arrangements (parent’s lap vs. chair). Our data show high consistency in attention to screen between participants, but likely achieved by different means. Infants showed considerable variability in the intensity of body movement and regulatory behaviours. Seating arrangements did not significantly affect infant behaviour. We found evidence for active dyadic physical contact throughout the session, and several parental behaviours, which may modulate infant looking. There was very limited evidence for sex differences in infant behaviour, and only for the duration of looking at the screen they reached statistical significance with boys looking somewhat longer than girls. Altogether, our coding scheme allows dynamical analyses of a range of infant behaviours in relation to looking at task performance, as well as exploring the social-interactive context of infant looking during standard eye-tracking procedures.

## Data Availability Statement

The datasets generated for this study can be obtained from the first author upon reasonable request via email.

## Ethics Statement

The studies involving human participants were reviewed and approved by the Departmental committee at the Faculty of Psychology, University of Warsaw, Poland. Written informed consent to participate in this study was provided by the participants’ legal guardian/next of kin.

## Author Contributions

PT conceived and supervised the project and data collection, wrote the first draft of the manuscript, finalised the manuscript, and conducted all the analyses. AM-K collected the data, coordinated the data collection and coding, and edited the manuscript.

## Conflict of Interest

The authors declare that the research was conducted in the absence of any commercial or financial relationships that could be construed as a potential conflict of interest.
