# Supplementary material for: What Do Young Infants Do During Eye-Tracking Experiments? IP-BET – A Coding Scheme for Quantifying Spontaneous Infant and Parent Behaviour
Source: Front Psychol. 2020 Apr 28;11:764. doi: 10.3389/fpsyg.2020.00764 (PMC7198886; doi:10.3389/fpsyg.2020.00764)
Supplement: Supplementary file 1 [file Data_Sheet_1.pdf]

Supplementary Material

What do young infants do during eye-tracking experiments? IP-BET – a coding scheme for quantifying spontaneous infant and parent behaviour

Przemysław Tomalski and Anna Malinowska-Korczak

1. Coding scheme categories and definitions

Table M1. Definitions and specifications of IP-BET coding scheme categories.

**Category**<br><br>Variable type; coding speed	**Subcategory**	**Definition**
**Infant Categories**		
**BODY MOVEMENT** Continuous, mutually exclusive, exhaustive<br><br>Coding speed 1x, two passes (1. code subcategories, 2. verify onset and offset).<br><br>Defined on the basis of the number of limbs or body parts moving at the same time. The intensity of an isolated movement is unimportant (e.g. how strongly infant bangs one arm). Each episode of a subcategory should last at least 1s. Two instances of the same subcategory should be separated by an episode lasting at least 2s. Movements of the head only are not coded (because they are related to attention shifting coded in another category).	**Immobility**	Total immobility of the body („freezing"; no head or body movement).
	**Low**	Minimal movements (e.g. moving fingers or wrist, shifting legs or arms from one position to another, hand waving).
	**Partial**	Movement of entire limbs or trunk, but not of entire body at once, e.g. one arm or both; one leg or both; trunk only; arms + legs but without trunk; arms + trunk; legs + trunk. Limb movement involves movement of the main joint: an elbow or a knee. Single movements within ankles or wrists are coded as „low body movement".
	**Full**	Movement of the entire body altogether (arms + legs + trunk).

**VISUAL ATTENTION**<br>Continuous<br>Coding speed 1/2x	**Looking at the screen**	Infant looking at the eye-tracker screen (also corners of the screen). Judged by the position of the face relative to the screen, not just the position of the eyes.
	**Looking towards the parent**	Infant visibly shifting head to look towards the parent.
	**Looking away from the screen**	Infant looking aside, upwards, directly at the camera placed above the screen, at own arms/legs or some object.
**OTHER BEHAVIOURS**<br>Coding speed 1x<br>Continuous except for pointing towards the screen, which is a point event	**Non-nutritive sucking**	Infant sucking/holding/manipulating hand, pacifier or other object placed in the mouth. Modifiers: pacifier or hand/object/other.
	**Self-touch**	Infant touching own body (e.g. head, rubbing hands together, holding own foot) or clothes (but only when visibly manipulating the fabric, holding open hand against it without manipulating is not coded as self-touch).
	**Object-related activity**	Infant actively touches an object or parent's clothes. Involves shifting, holding between fingers or in the hand, pulling or moving it. Just moving fingers placed on the object is not coded. Firm hold on the highchair's armrest is also coded. When the infant e.g. manipulates a pacifier for 3 sec and then stops, but the object is still held in the hand or the hand lies on the object – after the activity we use the Stop Code.
	**Pointing towards the screen (point event)**	Infant points in the direction of the screen.
**AFFECT**<br>Continuous<br>Coding speed 1x; each episode lasting at least 2 s	**Neutral (default)**	No clear sign of either positive or negative affect.
	**Positive**	Laughter; smile lasting at least 2 sec.
	**Negative**	Clear negative facial expression; a scream or a cry.

Parent		
**VISUAL ATTENTION**<br>Continuous<br>Coding speed 1x<br>Coding the object of parental focus of attention. Each episode (a look together with corresponding head movement) should last at least 2 s. Shorter glances are not coded.	**Looking at screen**	Parent's face directed towards the screen, can be looking or glancing at the baby at the same time.
	**Looking at infant**	Parent's face directed towards the baby and parent unable to look at the screen.
	**Looking away from the screen (elsewhere)**	Parent not looking at the screen or at the infant.
**OTHER BEHAVIOURS**<br>Point events<br>Coding speed 1x	**Pointing towards the screen**	Parent pointing towards a location on the screen.
	**Giving object to infant**	Parent giving an object to the infant, e.g. a pacifier, a toy or food.
	**Talking to infant**	Parent talking to the infant. Each phrase that has a new content or is said after a pause is coded as a separate event, e.g. „Look. Look, look at the ball" – coded as 1 instance; but phrase „look [~ 1 sec pause], look" is coded as two instances.
**Dyad**		
**DYADIC TOUCH**<br>Continuous<br>Coding speed 1x	Infant and parent are in physical contact, maintained actively, e.g. parent hugging the infant, holding infant's hand, infant visibly holding parent's hand. We also code physical contact when the parent is holding and stroking the infant. Active maintenance means that a hand passively lying on the arm of the parent does not constitute physical contact. Infant manipulating the parent's clothes does count in. When the infant is manipulating the parent's fingers for 3 sec then stopping, but the infant's hand remains in contact with the parent's hand (e.g. lying on it) use Stop Code.	
**INTERRUPTION**<br>Continuous<br>Coding speed 1x	Interrupting the progress of the eye-tracking session that results in the loss of ET data. Also, a situation, when another person is entering the testing room even when there was no ET data loss. No other events are coded during episodes of INTERRUPTION. Periods of Interruptions are excluded from the analysis.	

2. Coding scheme reliability data

Table S1. Inter-rater reliability (Cohen's kappas) for continuous exhaustive behaviours analysed as an agreement of sequence and duration calculated for pairs of coders.

Behavior category	Mean	SD	Minimum	Maximum
Infant movement	0.870	0.034	0.817	0.904
Infant visual attention	0.969	0.015	0.942	0.990
Infant behaviours - other (non-nutritive sucking, self-touch and object manipulation)	0.994	0.003	0.988	0.998
Infant affect	0.991	0.007	0.979	0.999
Parent visual attention	0.896	0.083	0.786	0.925
Dyadic Physical contact	0.896	0.083	0.739	0.925

3. Full descriptive data for coded categories

Table S2. Group averages for infant movement measures (entire sample seated on a lap, n=74; entire sample seated in a chair, n=20; SD in brackets).

**Measure**		**Lap - All**	**Lap - Girls**	**Lap - Boys**	**Chair - All**
Percentage of total observation time	Low movement	78.59 (14.40)	80.07 (12.13)	76.80 (16.84)	68.42 (19.59)
	Partial movement	19.97 (13.61)	19.01 (11.19)	21.05 (15.98)	30.97 (18.78)
	Full movement	1.44 (3.15)	0.92 (2.01)	2.51 (4.017)	0.61 (2.60)
No of infants (%) showing full movement		42 (56.8%)	22 (56.4%)	20 (58.8%)	2 (10%)
Average episode duration (sec)	Low movement	25.49 (21.84)	24.73 (22.16)	26.34 (21.77)	23.06 (16.43)
	Partial movement	4.82 (3.30)	4.27 (1.83)	5.43 (4.35)	8.51 (5.45)
	Full movement	1.74 (2.34)	1.58 (2.08)	1.91 (2.63)	0.45 (1.41)
Rate per minute	Low movement	2.59 (1.13)	2.70 (1.16)	2.47 (1.10)	2.22 (0.83)
	Partial movement	2.51 (1.14)	2.64 (1.15)	2.36 (1.12)	2.22 (0.85)
	Full movement	0.21 (0.40)	0.15 (0.23)	0.29 (0.52)	0.07 (0.29)

Table S3. Group averages for measures of infant looking at the screen, away from it and towards the parent (entire group seated on a lap, n=74, except for looking towards the parent, n=30; entire group seated in a chair, n=20, except for looking towards the parent n=8; SD in brackets).

**Measure**		**Lap - All**	**Lap - Girls**	**Lap - Boys**	**Chair - All**
Percentage of total observation time	Looking – screen	90.50 (4.72)	91.40 (4.39)	89.49 (4.94)	86.58 (6.67)
	Looking – away	9.26 (4.69)	8.33 (4.33)	10.30 (4.93)	12.51 (6.55)
	Looking – parent	0.59 (0.58)	0.63 (0.69)	0.55 (0.40)	2.21 (1.43)
No. of infants (%) looking towards parent		30 (40.5%)	17 (43.6%)	13 (37.1%)	8 (40%)
Average episode duration (sec)	Looking – screen	19.67 (18.36)	19.63 (10.32)	19.72 (24.59	14.04 (5.79)
	Looking – away	1.57 (0.61)	1.49 (0.60)	1.65 (0.62)	1.75 (0.63)
	Looking – parent	1.19 (0.67)	1.32 (0.80)	1.01 (0.40)	2.76 (3.17)
Rate per minute	Looking – screen	3.61 (1.37)	3.39 (1.31)	3.86 (1.41)	4.28 (1.70)
	Looking – away	3.54 (1.39)	3.34 (1.36)	3.77 (1.41)	4.19 (1.63)
	Looking – parent	0.30 (0.25)	0.29 (0.31)	0.31 (0.17)	0.62 (0.32)

Table S4. Frequencies and group averages for other infant behaviours (non-nutritive sucking, object-related activity and self-touch) and affect (data for neutral affect from the entire group seated on a lap, n=74, or in a chair, n=20; data for other categories calculated for infants showing these behaviours, SD in brackets).

**Measure**		**Lap - All**	**Lap - Girls**	**Lap - Boys**	**Chair - All**
Non-nutritive sucking	No. infants (%) showing	40 (54.5%)	17 (43.6%)	23 (65.7%)	11 (55%)
	No. infants (%) using pacifier	5 (6.8%)	3 (7.7%)	2 (5.9%)	4 (20%)
	Total duration as % time	19.97% (22.95%)	26.81% (27.14%)	14.91% (18.30%)	11.63% (17.73%)
	Mean episode duration (sec)	38.33 (73.72)	54.58 (102.32)	26.39 (40.84)	25.95 (56.24)
	Rate per minute	0.47 (0.39)	0.44 (0.41)	0.51 (0.38)	0.47 (0.44)
Object-related activity	No. infants (%) showing	21 (28.4%)	13 (33.3%)	8 (22.9%)	14 (70%)
	Total duration as % time	6.43% (10.03%)	7.50% (12.55%)	4,69% (3.44%)	8.19% (9.05%)
	Mean episode duration (sec)	10.20 (10.51)	12.69 (12.71)	6.14 (2.87)	7.80 (3.61)
	Rate per minute	0.34 (0.25)	0.27 (0.22)	0.45 (0.27)	0.62 (0.75)
Self-touch	No. infants (%) showing	46 (62.2%)	21 (53.8%)	25 (71.4%)	14 (70%)
	Total duration (% time)	6.20% (11.71%)	2.51% (3.75%)	9.29% (14.96%)	6.80% (12.89%)
	Mean episode duration (sec)	7.16 (11.93)	4.09 (2.03)	9.71 (15.76)	7.60 (11.86)
	Rate per minute	0.45 (0.51)	0.32 (0.34)	0.57 (0.60)	0.45 (0.45)
Neutral affect	Neutral - total duration (% time)	95.39% (12.66%)	97.55% (3.86%)	92.98% (17.77%)	96.38% (10.83%)
	Mean episode duration (sec)	295.83 (222.39)	353.31 (234.90)	231.78 (191.06)	460.20 (204.76)
Positive affect	No. infants (%) showing	28 (37.8%)	13 (33.3%)	15 (42.9%)	3 (15%)
	Positive total duration (% time)	3.25% (3.43%)	3.34% (3.90%)	3.18% (3.15%)	4.74 (3.98)
Negative affect	No. infants (%) showing	21 (29.7%)	10 (25.6%)	12 (34.3%)	3 (15%)
	Negative - total duration (% time)	7.17% (8.11%)	5.43% (4.15%)	8.75% (10.50%)	17.94% (26.42%)

Table S5. Frequencies and group averages for measures of parental interactive behaviours (talking to the infant, pointing to the screen) and parental looking (at the screen, away from it and at the infant). Data on looking for the entire sample of infants seated on a parent's lap, n=74, or in a chair, n=20; data for frequency of talking and pointing only for parents showing these behaviours, SD in brackets.

**Measure**		**Lap - All**	**Lap - Girls**	**Lap - Boys**	**Chair - All**
Parent talking to infant	No parents (%) showing	34 (45.9%)	19 (48.7%)	15 (42.9%)	2 (10%)
	Rate per minute (for those showing)	1.21 (1.63)	1.35 (2.04)	1.04 (0.93)	1.32 (1.10)
Parent pointing to the screen	No parents (%) showing	19 (25.7%)	10 (28.6%)	9 (23.1%)	7 (35%)
	Rate per minute (for those showing)	0.50 (0.45)	0.55 (0.61)	0.45 (0.28)	0.62 (0.87)
Percentage of total observation	Looking at the screen	75.44% (29.40%)	75.37% (28.60%)	75.53% (30.68%)	63.68% (26.31%)
	Looking away from the screen	4.04% (15.36%)	4.68% (16.04%)	3.33% (14.75%)	12.35% (20.55%)
	Looking at the infant	20.40% (26.52%)	19.74% (24.82%)	21.15% (28.65%)	15.91% (18.26%)
Average episode duration (sec)	Looking at the screen	69.54 (83.55)	65.21 (78.56)	74.38 (89.69)	40.03 (40.34)
	Looking away from the screen	7.19 (29.46)	6.12 (12.75)	8.38 (40.98)	8.05 (11.36)
	Looking at the infant	29.58 (104.71)	23.32 (92.12)	36.54 (118.15)	7.32 (7.86)
Rate per minute	Looking at the screen	1.30 (1.05)	1.42 (1.18)	1.17 (0.88)	2.11 (2.78)
	Looking away from the screen	0.20 (0.29)	0.20 (0.24)	0.20 (0.34)	0.48 (0.46)
	Looking at the infant	1.13 (1.01)	1.28 (1.12)	0.97 (0.84)	1.61 (2.96)

Table S6. Frequencies and group average data for episodes of dyadic physical contact (data only for dyads showing these behaviours, SD in brackets).

**Measure**		**Lap - All**	**Lap - Girls**	**Lap - Boys**	**Chair - All**
Dyadic physical contact	No dyads (%) showing	64 (86.5%)	31 (88.6%)	33 (84.6%)	9 (75%)
	Total duration (% time)	18.37% (22.63%)	19.83% (26.17%)	16.83% (18.46%)	19.83% (29.61%)
	Mean episode duraiton (sec)	23.93 (74.08)	34.17 (101.17)	13.03 (19.73)	53.43 (121.10)
	Rate per minute	0.86 (0.67)	0.82 (0.73)	0.91 (0.61)	0.43 (0.34)

Table S7. Correlations of total duration of infant partial and full movement with infant looking at the screen or away from it (Pearson's *r* and *p* values). Significant results are marked with asterisks, **p < .01 level (2-tailed).

		1.	2.	3.	4.	5.	6.	7.
1. Partial and full movement (total duration)	*r*	1	-.396**	-.187	.362**	.401**	.142	.372**
	p		0.000	0.110	0.002	0.000	0.229	0.001
2. Infant looking - screen (total duration)	*r*	-.396**	1	.546**	-.709**	-.995**	-.642**	-.721**
	p	0.000		0.000	0.000	0.000	0.000	0.000
3. Infant looking - screen (mean duration)	*r*	-.187	.546**	1	-.675**	-.537**	-.111	-.676**
	p	0.110	0.000		0.000	0.000	0.346	0.000
4. Infant looking - screen (rpm)	*r*	.362**	-.709**	-.675**	1	.702**	-.006	.995**
	p	0.002	0.000	0.000		0.000	0.957	0.000
5. Infant looking - away (total duration)	*r*	.401**	-.995**	-.537**	.702**	1	.654**	.710**
	p	0.000	0.000	0.000	0.000		0.000	0.000
6. Infant looking - away (mean duration)	*r*	.142	-.642**	-.111	-.006	.654**	1	.002
	p	0.229	0.000	0.346	0.957	0.000		0.990
7. Infant looking - away (rpm)	*r*	.372**	-.721**	-.676**	.995**	.710**	.002	1
	p	0.001	0.000	0.000	0.000	0.000	0.990	
